# Feasibility of Measuring Sedentary Time Using Data From a Thigh-Worn Accelerometer: The 1970 British Cohort Study

**DOI:** 10.1093/aje/kwaa047

**Published:** 2020-03-27

**Authors:** Mark Hamer, Emmanuel Stamatakis, Sebastien Chastin, Natalie Pearson, Matt Brown, Emily Gilbert, Alice Sullivan

**Keywords:** cohort studies, lifestyle, physical activity, population studies, sedentary time, sitting, wearable devices

## Abstract

In large-scale cohort studies, sedentary behavior has been routinely measured using self-reports or devices that apply a count-based threshold. We employed a gold standard postural allocation technique using thigh inclination and acceleration to capture free-living sedentary behavior. Participants aged 46.8 (standard deviation (SD), 0.7) years (*n* = 5,346) from the 1970 British Cohort Study (United Kingdom) were fitted with a waterproofed thigh-mounted accelerometer device (activPAL3 micro; PAL Technologies Ltd., Glasgow, United Kingdom) worn continuously over 7 days; data were collected in 2016–2018. Usable data were retrieved from 83.0% of the devices fitted, with 79.6% of the sample recording at least 6 full days of wear (at least 10 waking hours). Total daily sitting time (average times were 9.5 (SD, 2.0) hours/day for men and 9.0 (SD, 2.0) hours/day for women) accounted for 59.4% and 57.3% of waking hours in men and women, respectively; 73.8% of sample participants recorded ≥8 hours/day of sitting. Sitting in prolonged bouts of 60 continuous minutes or more accounted for 25.3% and 24.4% of total daily sitting in men and women, respectively. In mutually adjusted models, male sex, underweight, obesity, education, poor self-rated health, television-viewing time, and having a sedentary occupation were associated with higher device-measured sitting times. Thigh-worn accelerometry was feasibly deployed and should be considered for larger-scale national surveys.

## Abbreviations

CI: confidence interval
SD: standard deviation
TV: television

Sedentary behavior has been recognized as a risk factor for poor health (1–5). However, to date, the evidence generated from large-scale population cohort studies has relied on self-reporting, a method with known biases (6). For example, data from the National Health and Nutrition Examination Survey suggested that total self-reported sitting time in adults increased from 5.5 hours/day to 6.4 hours/day during 2007–2016 (7) and showed that 25.7% of US adults reported more than 8 hours of total sitting time per day (8). Given the uncertainty regarding the validity of data on self-reported sitting time, it is difficult to estimate true population norms for sitting (9, 10).

Wearable devices are being increasingly used to assess free-living sedentary time (11, 12), although most of the existing methods have applied criteria based on lack of movement or movement below a certain count threshold (13). The count-based threshold approach, which applies cutpoints to classify movement intensity, can lead to misclassification of low-intensity nonsedentary behaviors such as standing (14–16). Thus, ideally measurements of sedentary time should be derived from a combination of both energy expenditure and postural elements (12).

In the present study, we sought to use a thigh-mounted accelerometer, the activPAL3 micro device (PAL Technologies Ltd., Glasgow, United Kingdom), to assess sedentary behavior (12). The device uses derived information about thigh inclination and acceleration to estimate body posture (i.e., sitting/lying and upright) and transition between these postures, stepping, and stepping speed (cadence). Importantly, this technique overcomes concerns raised (14–16) about the face validity of wrist- and hip-worn monitors to accurately capture postural sitting. ActivPAL was validated for measuring free-living sedentary behavior against direct observation using an automated camera (17). Although the thigh-mounted accelerometer has been used in relatively small convenience samples thus far (12, 18), the present study is the first (to our knowledge) to have used this approach in a large general population sample. Such studies are important to explore the feasibility of deploying a potentially more invasive device attached to the skin (compared with devices worn around the wrist or attached to waist belts) so that this methodology could be considered for larger-scale national surveys in the future.

Our aim in this study was to explore the feasibility of using a gold standard postural allocation technique to capture free-living sedentary behavior in a large nationally representative cohort study of middle-aged British adults. We report on rates of consent and adherence to the device-wear protocol. Our second aim was to examine sociodemographic and lifestyle correlates of free-living sitting.

## METHODS

### Design and participants

The 1970 British Cohort Study recruited participants from England, Scotland, and Wales who were born during a single week in 1970 (19, 20). The cohort has been followed up with regular assessments throughout childhood and adulthood. The age 46 years survey was a home visit conducted in 2016–2018 and comprised 50 minutes of interviews (both face-to-face computer-assisted personal interviews and computer-assisted self-completion interviews), with a further 50 minutes of biomedical assessments performed by trained nurses (19). Participants provided informed consent, and the study received full ethical approval from the NRES Committee South East Coast–Brighton and Sussex.

### Sedentary behavior measurement

The study used a thigh-mounted accelerometer (the activPAL3 micro device) as previously described (12). We utilized a wear protocol that had been previously adopted (18). Devices were programmed to sample at the default frequency of 20 Hz. The device was waterproofed and fitted by a trained nurse on the midline anterior aspect of the upper thigh, as recommended by the manufacturer. Participants were requested to wear the device continuously for 7 days, including sleeping, bathing, swimming, and all physical activities. If the device fell off or was removed before the stated end date, participants were requested not to reattach it. Devices were returned via mail. Data were processed using freely available software that had been previously validated (21). The software uses an algorithm to isolate valid waking wear data and distinguish it from periods of sleep or prolonged nonwear. The algorithm rules have been summarized elsewhere (21). Briefly, bouts of sleep/nonwear were identified as 1) the longest bout per 24-hour period (from noon to noon each day) that lasted ≥2 hours or 2) any very long bouts lasting ≥5 hours. This permits sleep/nonwear to occur at any time, any number of times (including never) within a 24-hour window. Since sleep can register as multiple periods of sitting/lying interspersed with real or erroneously detected posture changes and stepping, the next step iteratively examined surrounding bouts and determined whether they were more likely to be additional sleep/nonwear (limited movement) or waking wear (more movement). Bouts were deemed “surrounding” if any portion was within a 15-minute window before or after a bout of sleep/nonwear. All bouts in the sleep window were classed as sleep/nonwear when the window contained any of the following: a long bout (>2 hours) of sitting/lying or standing or a moderately long bout (≥30 minutes) with very few (≤20) steps in between; a bout of sleeping/nonwear; or posture changes without intervening steps. This step was repeated until no further sleep/nonwear was found. We defined “sleep” more broadly as the period a participant spent in bed, from the time of getting “into bed” or “lights out” to the time of finally awakening or arising from bed, including brief periods out of bed such as trips to the bathroom. Importantly, the algorithm was not designed to provide subclassifications of the excluded data, such as sleep versus nonwear, or time spent asleep by biological definitions versus other time spent in bed. We used a step cadence threshold of ≥100 to derive moderate- to vigorous-intensity physical activity (22). The first partial day was removed, and subsequent days were defined from midnight to midnight. Participants were included if they recorded data on at least 1 valid day during the monitoring period, defined as at least 10 hours of waking wear time. Participants were also administered sleep diaries that were completed on a daily basis concurrently with wearing the device.

Participants provided self-reported data on context-specific sedentary behaviors, including television (TV)-viewing, Internet use, video games, reading (categorical: none, <1.0 hour/day, 1.0–1.9 hours/day, 2.0–2.9 hours/day, 3.0–3.9 hours/day, 4.0–4.9 hours/day, or ≥5.0 hours/day), and automobile use. Data on occupational activity were derived from a combination of questions on predominant activity at work (sitting, standing, physical labor, or heavy manual labor) and information on social occupational group (professional; intermediate; lower supervisory/technical; semiroutine/routine; long-term unemployed).

### Lifestyle and health measures

Participants provided information on smoking habits (categorical: never smoker, ex-smoker, or current smoker), self-rated health (categorical: excellent, very good, good, fair, or poor), disability (using the European Statistics on Income and Living Conditions classification (categorical): none, to some extent, or severely hampered), and education (categorical: none, General Certificate of Secondary Education/General Certificate of Education Advanced Level/diploma (high school), or university degree). Nurses measured height and weight for the calculation of body mass index (weight (kg)/height (m)^2^), which was categorized as underweight (<18.5), normal-weight (18.5–24.9), overweight (25.0–25.9), obese (30.0–34.9), or morbidly obese (≥35.0). Blood pressure was measured using an automated Omron HEM-907 device (Omron Corporation, Kyoto, Japan) after 5 minutes of seated rest. Participants provided information on physician-diagnosed hypertension and diabetes. Hypertension was defined by physician diagnosis and/or elevated systolic (≥140 mm Hg) and diastolic (≥90 mm Hg) blood pressure.

### Statistical analyses

The distributions of activPAL variables were examined for normality and potential outliers. The activity data represented mean hours per day averaged over the number of days on which the device was worn. Extreme waking hours of wear time (*n* = 13; >20 hours/day) were checked against sleep diaries, and in the case of clear discrepancies (±3 standard deviations (SDs)), outliers were removed. The acceptability of the device (in terms of consent to participate and number of days worn) was examined in relation to sociodemographic characteristics. Total sitting time was categorized into tertiles (low: <8.4 hours/day; medium: 8.4–10.1 hours/day; and high: >10.1 hours/day) and examined in relation to sociodemographic and lifestyle variables. We also derived data on bouts of uninterrupted sitting time lasting 60 minutes or more. The sociodemographic and lifestyle variables were selected a priori on the basis of existing literature (23, 24). Generalized linear models were used to examine associations of sociodemographic and lifestyle variables with sitting time as a continuous dependent variable, with adjustment for waking hours of wear time.

**Table 1:** Descriptive Data^a^ Obtained From the ActivPAL3micro Accelerometer^b^ (*n* = 5,346), 1970 British Cohort Study, 2016–2018

**Variable**	**Men** **(*n* = 2,542)**	**Women** **(*n* = 2,804)**
Total waking device wear time, hours/day	15.9 (1.3)	15.7 (1.3)
No. of days of device wear	6.1 (1.6)	6.2 (1.5)
Sitting time, hours/day	9.5 (2.0)	9.0 (2.0)
Prolonged sitting (≥60 minutes), hours/day	2.4 (1.5)	2.2 (1.4)
Standing time, hours/day	4.4 (1.5)	4.7 (1.5)
Total activity, hours/day	2.0 (0.8)	2.0 (0.7)
MVPA, minutes/day	50.4 (24)	51.6 (24)

Abbreviation: MVPA, moderate–vigorous physical activity.

^a^ Values are expressed as mean (standard deviation).

^b^ PAL Technologies Ltd., Glasgow, United Kingdom.

**Table 2:** Characteristics (%) of Participants in Relation to Daily Sitting Time, 1970 British Cohort Study, 2016–2018

	**Level of Sitting Time**		
**Variable**	**Low ($ \boldsymbol{<}$8.4 Hours/Day)** **(*n* = 1,791)**	**Medium (8.4–10.1 Hours/Day)** **(*n* = 1,780)**	**High ($ \boldsymbol{>}$10.1 Hours/Day)** **(*n* = 1,775)**
Age, years^a^	46.8 (0.7)	46.8 (0.7)	46.8 (0.7)
Sex			
Male	40.0	46.6	56.1
Female	60.0	53.4	43.9
Smoking status			
Never smoker	49.2	50.5	48.4
Past smoker	32.7	33.1	32.3
Current smoker			
Occasional smoker	5.1	4.8	4.2
Daily smoker	13.0	11.6	15.1
Education			
None	30.0	23.7	24.6
High school	48.5	45.2	44.4
College degree	21.5	31.1	31.0
Self-rated health			
Excellent	19.9	19.6	18.2
Very good	37.9	39.5	34.5
Good	28.3	27.0	27.4
Fair	12.0	10.4	13.5
Poor	1.9	3.5	6.4
Disability			
None	86.3	85.6	81.9
Some	11.1	9.4	10.9
Severe	2.6	5.0	7.2
BMI^b^ category			
Underweight (<18.5)	1.1	0.4	0.7
Normal-weight (18.5–24.9)	35.5	29.7	23.4
Overweight (25.0–25.9)	36.4	39.8	39.3
Obese (30.0–34.9)	24.5	27.8	32.2
Morbidly obese (≥35.0)	2.5	2.3	4.4
Physician-diagnosed diabetes	1.8	1.5	3.8
Physician-diagnosed hypertension	6.6	6.2	8.9
MVPA, hours/day^a^	1.0 (0.5)	0.8 (0.4)	0.7 (0.3)
No. of days of device wear^a^	6.1 (1.7)	6.3 (1.4)	6.1 (1.6)
TV-viewing time, hours/day			
<1.0	18.6	16.0	13.0
1.0–1.9	38.6	37.1	32.4
2.0–2.9	27.7	28.5	29.1
≥3.0	15.1	18.4	25.5
Video game time, hours/day			
0 (none)	73.4	70.7	61.6
1 hour/day	16.4	18.1	22.2
≥1 hour/day	10.2	11.2	16.2
Internet use, hours/day			
<1.0	41.9	36.0	33.4
1.0–1.9	38.8	39.2	40.7
2.0–2.9	11.7	14.6	12.3
≥3.0	7.6	10.2	13.6
Reading time, hours/day			
0 (none)	39.5	37.9	35.5
<1	45.4	46.6	44.7
≥1	15.1	15.5	19.8
Car use for short journeys (<5 miles (<8 km))	75.8	78.1	79.7
Occupational activity			
Sitting	26.7	54.8	68.0
Standing	23.0	12.7	7.0
Physical labor	35.1	21.2	12.6
Heavy manual labor	7.8	2.7	2.4
Unemployed	7.4	8.6	10.0

Abbreviations: BMI, body mass index; MVPA, moderate–vigorous physical activity; TV, television.

^a^ Values are expressed as mean (standard deviation).

^b^ Weight (kg)/height (m)^2^.

## RESULTS

Usable data were retrieved from 83.0% of the devices fitted (see Web Figure 1, available at https://academic.oup.com/aje). Persons who declined to wear the device (11.8%) were more likely to be male, to be smokers, to report poorer health, and to be obese (Web Table 1). Reasons for declining to wear the device mainly included “inconvenience” and “going on holiday” (planning to travel by plane was an exclusion criterion), while relatively few had concerns over attachment of the device to the skin (Web Figure 1).

The final analytical sample comprised 5,346 men and women aged 46.8 (SD, 0.7) years. We observed high adherence to the wear protocol: 90.7% of the sample recorded at least 3 full days of device wear, 79.6% recorded 6 full days of wear, and 65.5% wore the device for the full 7 days. Compared with participants with higher wear adherence (>3 days), those with poor adherence (≤3 days) were more likely to be male, to be smokers, to report poorer health, to be obese, and to not have a college degree (Web Table 2). Interestingly, those with poor adherence were more likely to have worn the device over the summer months. Nevertheless, no differences were observed between the groups for average sitting time or activity.

Total daily sitting time was normally distributed (Web Figure 2) (average times were 9.5 (SD, 2.0) hours/day for men and 9.0 (SD, 2.0) hours/day for women (Table 1)) and accounted for 59.4% and 57.3% of waking hours in men and women, respectively. Overall, 73.8% of the sample recorded ≥8 hours/day of sitting. Sitting in prolonged bouts of 60 continuous minutes or more accounted for 25.3% and 24.4% of total daily sitting in men and women, respectively (Web Figure 3).

Participants recording higher sitting times were more likely to be male, smokers, college-educated, and obese and to report higher prevalences of poor health and disability, hypertension, and diabetes (Table 2). There was a trend toward higher prevalence of all self-reported sedentary behaviors in the highest device-measured sitting time group (Table 2). The differences were particularly noticeable for TV-viewing and sedentary occupations. Correlations between various self-reported sedentary behaviors and device-measured daily sitting time were as follows: for TV-viewing, *r* = 0.15 (*P* < 0.001); for Internet use, *r* = 0.12 (*P* < 0.001); for reading, *r* = 0.06 (*P* < 0.001); and for occupational sitting, *r* = 0.36 (*P* < 0.001). In generalized linear models with results mutually adjusted for all variables, sex, underweight, obesity, education, self-rated health, TV-viewing time, and having a sedentary occupation remained independently associated with device-measured sitting time (Table 3). In particular, participants in sedentary occupations recorded 2.00 hours/day (95% confidence interval (CI): 1.80, 2.27) more sitting than persons in heavy manual labor occupations; participants reporting ≥3 hours/day of TV-viewing recorded 0.89 hours/day (95% CI: 0.71, 1.07) more sitting time than participants reporting <1 hour/day of TV-viewing; and the morbidly obese recorded an extra 0.88 hours/day (95% CI: 0.59, 1.18) of sitting compared with normal-weight individuals. We repeated these analyses for sitting time recorded in bouts of 60 minutes or more (Table 4). The results remained largely unchanged, except that seasonal differences emerged showing less prolonged sitting in the spring (β = −0.16, 95% CI: −0.26, −0.07) and summer (β = −0.15, 95% CI: −0.25, −0.05) than in the winter.

**Table 3:** Sociodemographic and Lifestyle Factors Associated with Accelerometer-Measured Sitting Time (Hours/Day), 1970 British Cohort Study, 2016–2018

**Variable**	**No. of** **Persons**	**β** ^a^	**95% CI**
Sex			
Male	2,542	0	Referent
Female	2,804	−0.36	−0.46, −0.25
Education			
None	1,405	0	Referent
High school	2,461	0.17	0.05, 0.29
College degree	1,480	0.55	0.42, 0.69
Smoking status			
Never smoker	2,648	0	Referent
Ex-smoker	1,748	0.03	−0.08, 0.14
Current smoker	950	0.07	−0.08, 0.24
Self-rated health			
Excellent	1,022	0	Referent
Very good	1,997	0.02	−0.12, 0.16
Good	1,478	0.05	−0.10, 0.20
Fair	637	0.11	−0.08, 0.31
Poor	212	0.96	0.63, 1.29
Disability			
None	4,521	0	Referent
Some extent	561	0.06	−0.11, 0.22
Severely hampered	264	0.76	0.46, 1.04
BMI^b^ category			
Normal-weight (18.5–24.9)	1,565	0	Referent
Underweight (<18.5)	109	0.51	0.16, 0.87
Overweight (25.0–25.9)	2,032	0.28	0.16, 0.40
Obese (30.0–34.9)	1,479	0.43	0.30, 0.57
Morbidly obese (≥35.0)	161	0.88	0.59, 1.18
Occupation			
Heavy manual labor	230	0	Referent
Sitting	2,660	2.00	1.80, 2.27
Standing	762	0.46	0.19, 0.72
Physical labor	1,230	0.40	0.14, 0.64
Unemployed	464	1.58	1.30, 1.85
TV-viewing time, hours/day			
<1.0	834	0	Referent
1.0–1.9	1,890	0.23	0.08, 0.38
2.0–2.9	1,488	0.54	0.34, 0.70
≥3.0	1,134	0.89	0.71, 1.07
Month of data collection			
Winter	1,968	0	Referent
Spring	1,383	−0.09	−0.22, 0.03
Summer	1,020	−0.12	−0.25, 0.02
Autumn	975	−0.06	−0.20, 0.06

Abbreviations: BMI, body mass index; CI, confidence interval; TV, television.

^a^ Beta (β) coefficients were mutually adjusted for all other variables shown in the table and for waking hours of device wear time.

^b^ Weight (kg)/height (m)^2^.

**Table 4:** Sociodemographic and Lifestyle Factors Associated with Acceleromater-Measured Bouts of Time Spent Sitting for 60 Minutes or More (Hours/Day), 1970 British Cohort Study, 2016–2018

**Variable**	**No. of Persons**	**β** ^a^	**95% CI**
Sex			
Male	2,542	0	Referent
Female	2,804	−0.11	−0.19, −0.03
Education			
None	1,405	0	Referent
High school	2,461	0.04	−0.05, 0.14
College degree	1,480	0.19	0.09, 0.30
Smoking status			
Never smoker	2,648	0	Referent
Ex-smoker	1,748	−0.11	−0.20, −0.03
Current smoker	950	−0.39	−0.51, −0.27
Self-rated health			
Excellent	1,022	0	Referent
Very good	1,997	0.05	−0.06, 0.15
Good	1,478	0.13	0.01, 0.24
Fair	637	0.18	0.03, 0.33
Poor	212	0.98	0.72,1.23
Disability			
None	4,521	0	Referent
Some extent	561	0.06	−0.07, 0.19
Severely hampered	264	0.65	0.44, 0.87
BMI^b^ category			
Normal-weight (18.5–24.9)	1,565	0	Referent
Underweight (<18.5)	109	0.57	0.30, 0.84
Overweight (25.0–25.9)	2,032	0.15	0.06, 0.25
Obese (30.0–34.9)	1,479	0.41	0.31, 0.51
Morbidly obese (≥35.0)	161	0.87	0.64, 1.09
Occupation			
Heavy manual labor	230	0	Referent
Sitting	2,660	0.56	0.36, 0.74
Standing	762	0.01	−0.19, 0.22
Physical labor	1,230	−0.02	−0.21, 0.17
Unemployed	464	0.64	0.41, 0.87
TV-viewing time, hours/day			
<1.0	834	0	Referent
1.0–1.9	1,890	0.11	0.002, 0.23
2.0–2.9	1,488	0.30	0.18, 0.42
≥3.0	1,134	0.58	0.44, 0.71
Month of data collection			
Winter	1,968	0	Referent
Spring	1,383	−0.16	−0.26, −0.07
Summer	1,020	−0.15	−0.25, −0.05
Autumn	975	−0.08	−0.19, 0.02

Abbreviations: BMI, body mass index; CI, confidence interval; TV, television.

^a^ Beta (β) coefficients were mutually adjusted for all other variables shown in the table and for waking hours of device wear time.

^b^ Weight (kg)/height (m)^2^.

## DISCUSSION

Given the importance of obtaining accurate population measures of health behaviors for informing health policies, it is crucial to understand the feasibility of introducing novel wearable technology on a large scale. In this study, we demonstrated the feasibility of using a thigh-worn accelerometer to capture free-living sedentary behavior in a large nationally representative cohort of middle-aged British adults. The thigh-worn accelerometer has demonstrated superiority over other devices for measuring changes in daily sitting time. For example, Kozey-Keadle et al. (16) showed that in comparison with the thigh-worn activPAL, sensitivities for the waist-worn ActiGraph 100 cutpoint and the ActiGraph 150 cutpoint were 80% (95% CI: 50, 100) and 70% (95% CI: 43, 97), respectively. Specificity was 67% (95% CI: 39, 94) for both.

Despite attrition, the present birth cohort sample remains broadly representative (25) and characterizes some of the features of contemporary Western society, including high prevalences of obesity and inactivity. The majority of cohort members (88.2%) who were approached to wear the device agreed to participate. Usable data were retrieved from 83.0% of the devices placed; 79.6% and 65.5% recorded at least 6 and 7 full days of wear, respectively. Our wear data were largely comparable with those from smaller activPAL studies (e.g., the SeniorsUSP Study (*n* = 773; 91% with 7 days' valid wear), the Walking Away From Type 2 Diabetes Study (*n* = 530; 67% with 7 days' valid wear), and the Australian Diabetes, Obesity and Lifestyle Study (*n* = 782; 79% with 7 days' valid wear) (12, 18). Differences in wear compliance might be explained by differences in the characteristics of the samples and/or study protocols. For example, although we largely replicated the SeniorsUSP Study protocol (18), a key difference was a reliance on our participants to return their devices via mail, as compared with monitors’ being removed by researchers in the SeniorsUSP Study. In addition, the SeniorsUSP Study consisted of older participants.

Our data were also comparable with large-scale population data on wrist-worn accelerometry in British adults; for example, investigators in the UK Biobank Study reported 93.3% of usable data from devices placed, and 80.6% of participants wore the device for at least 150 hours (approximately 6 days) out of a scheduled 168 hours (26). Season appeared to have influenced wear compliance in our study (lower in summer months), although no seasonal wear-time differences were reported in the UK Biobank Study (26). In addition, participants recorded greater prolonged bouts of sitting in the winter.

One of the most striking features of the present study was the markedly higher proportion of participants recording more than 8 hours of total sitting time per day as compared with previous population estimates from self-reports. For example, National Health and Nutrition Examination Survey data showed that only 25.7% of US adults reported more than 8 hours of total self-reported sitting time per day (8), while our data suggested that 73.8% of our sample reached this threshold. Interestingly, data on self-reported sedentary behaviors were far more comparable; for example, 62% of adults in the National Health and Nutrition Examination Survey sample reported >2 hours/day of TV-viewing/video games (7), as compared with 53% of the present British sample. Overall, this reflects the difficulties of recalling total daily sitting time.

Total sitting time was socially patterned—higher in college-educated participants. This finding is likely to be partly driven by occupation, in that professionals/managers are most likely to be desk-bound at work. In contrast, however, previous data suggested that lower-social-status groups report greater sedentary behavior, such as TV-viewing, during their leisure time (23). Thus, social patterning of sedentary behavior is likely to be context-specific (not simply volume-related), although our data suggest it is largely driven by occupational sitting time. The other correlates of device-measured sitting time, such as obesity and health indicators, found in this study were also consistent with other studies on self-reported TV-viewing (24).

The main strengths of this study were the nationally representative sample and the high level of adherence to the wear protocol, with little data loss. Our wear protocol minimized the problems of nonwear, as participants were requested not to reattach the device if it was removed prematurely, as was done previously (18). We employed a novel algorithm to isolate valid waking wear time from sleep/nonwear time that enabled large volumes of accelerometry data to be processed more efficiently.

This study also had limitations. As is the case in most population studies, respondents who did not consent to wear a device tended to be less educated and to report poorer health, which may have introduced bias. Participants with greater compliance in wearing the device were also generally healthier, although device wear characteristics did not appear to influence the amount of sitting or activity recorded. Wearable activity monitors are generally designed to be worn for no more than 1 week in order to minimize participant burden; this may not adequately reflect habitual behavior. Since our study was conducted in middle-aged adults, before the onset of functional decline, the results may not be representative of the wider population. The algorithm was not designed to distinguish physiological sleep periods, and in the absence of a true “gold standard” we were unable to more fully explore sleep in this study. Data were cross-sectional, and this limits interpretation on the directionality of the associations between sedentary time and demographic characteristics. Our measure of sedentary behavior was an average of weekday and weekend activity, and participants contributed differential wear times to this average. Nevertheless, average sitting times of participants with a full 7 days of wear were identical to data from the whole cohort.

In summary, thigh-worn accelerometers can be feasibly deployed in large-scale population cohort studies. Future studies should be mindful of potential selection biases when using wearable technology, in terms of both consent to participate and wear compliance.

## Supplementary Material

kwaa047_Hamer_Web_Material_Final
