# Supplementary material for: Feasibility of Measuring Sedentary Time Using Data From a Thigh-Worn Accelerometer: The 1970 British Cohort Study
Source: Am J Epidemiol. 2020 Mar 27;189(9):963–71. doi: 10.1093/aje/kwaa047 (PMC7443760; doi:10.1093/aje/kwaa047)

Web Material

Feasibility of Measuring Sedentary Time Using Data From a Thigh-Worn Accelerometer: The 1970 British Cohort Study

Web Table 1
Web Table 2
Web Figure 1
Web Figure 2
Web Figure 3

Web Table 1. Comparison of respondents willing to wear the activity monitor with those who refused

	Consented to wear device	Non-consent
N	6563	877
Men (%)	47.7	55.0
Smokers (%)	14.6	18.2
Degree educated (%)	22.8	19.3
Poor self-rated health (%)	4.8	6.6
Disability* (%)	5.6	9.3
Obese (% ≥ 30 kg/m^2)	31.6	32.5
Seasonality (%)		
Winter	37.6	32.8
Spring	24.6	22.6
Summer	20.1	22.6
Autumn	17.7	22.0
Occupation (%)		
Sitting	54.3	58.8
Standing	15.5	14.8
Physical work	25.4	21.9
Heavy manual	4.8	4.6

*Disability classification EU-SILC, severely hampered

Web Table 2. Comparison of respondents in relation to wear period

	≤ 3 full days	>3 full days
Men (%)	51.3	46.8
Smokers (%)	15.4	13.5
Degree educated (%)	20.1	24.2
Poor self-rated health (%)	7.0	3.6
Disability* (%)	8.0	4.6
Obese (% ≥ 30 kg/m^2)	40.2	29.7
Sitting time (hr/d)	9.3± 2.5	9.2± 1.9
Activity time (hr/d)	2.0± 0.9	2.0± 0.7
Waking wear time (hr/d)	15.7± 1.9	15.9± 1.1
Season		
Winter	31.4	38.3
Spring	20.5	26.2
Summer	26.4	17.9
Autumn	21.7	17.6

*Disability classification EU-SILC, severely hampered.

Web Figure 1. Flow of participants into the BCS70 activPAL3 micro study

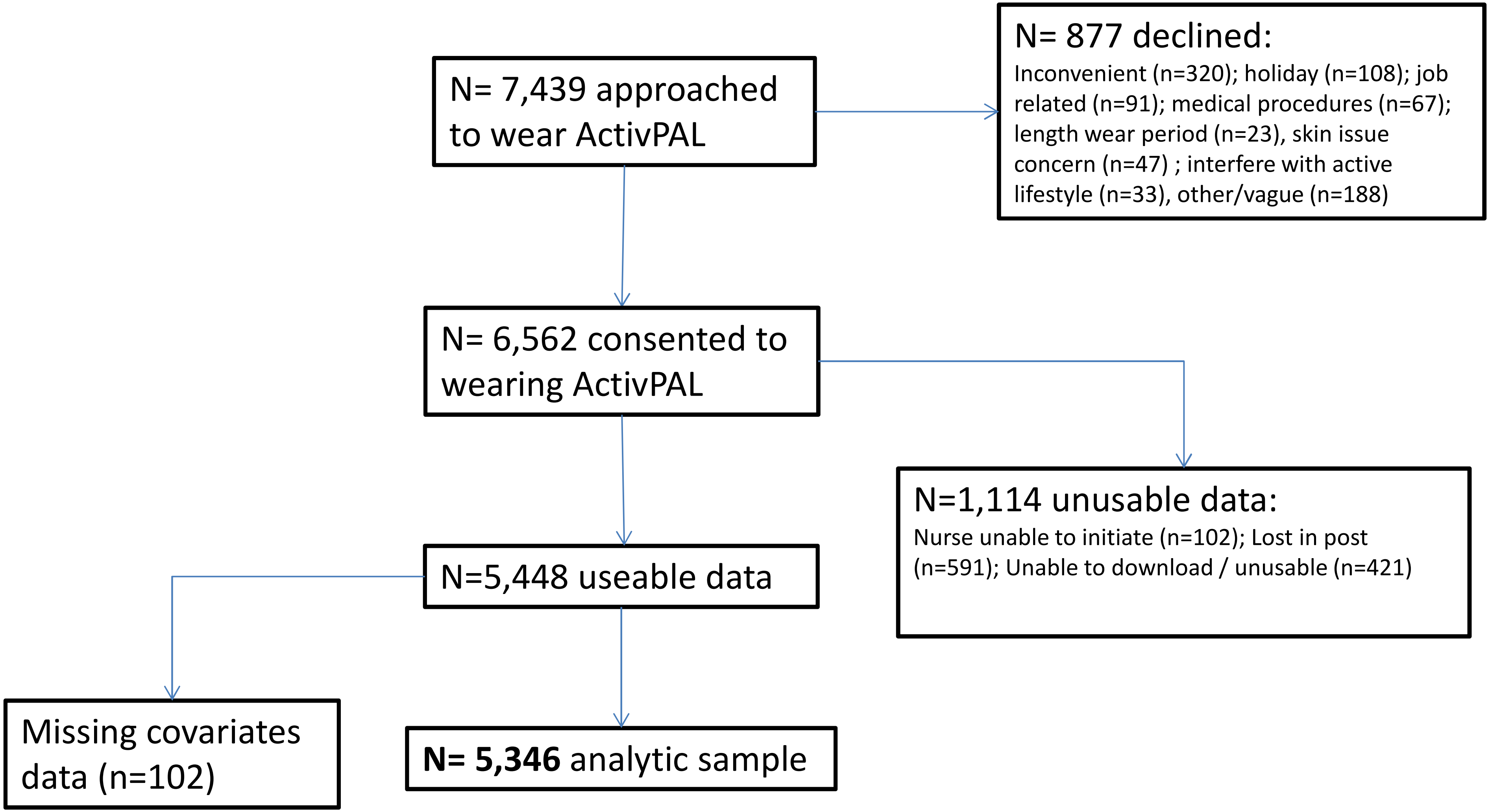

Web Figure 2. Distribution of device-measured sitting time

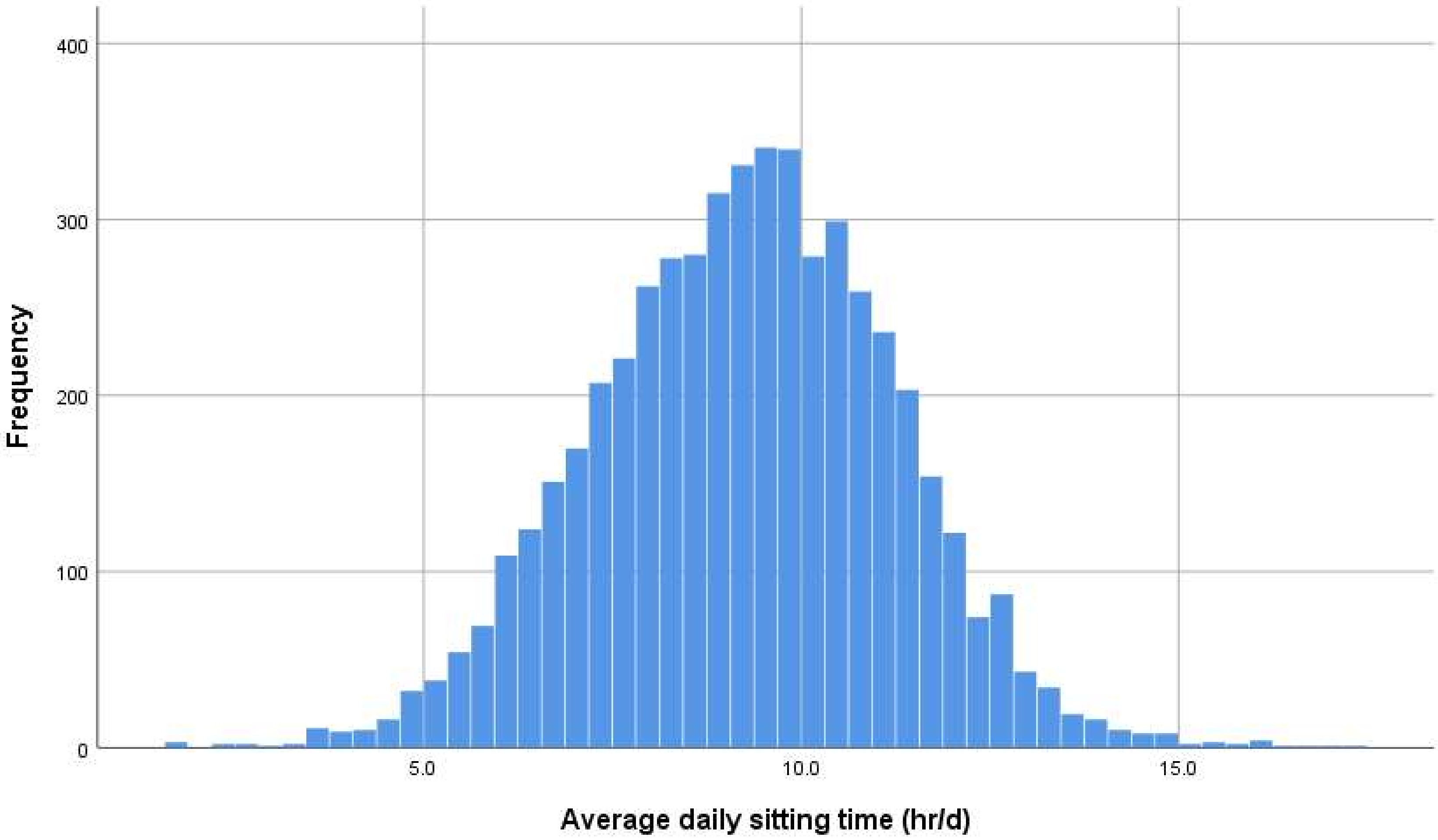

Web Figure 3. Distribution of prolonged bouts of sitting (60+ min)

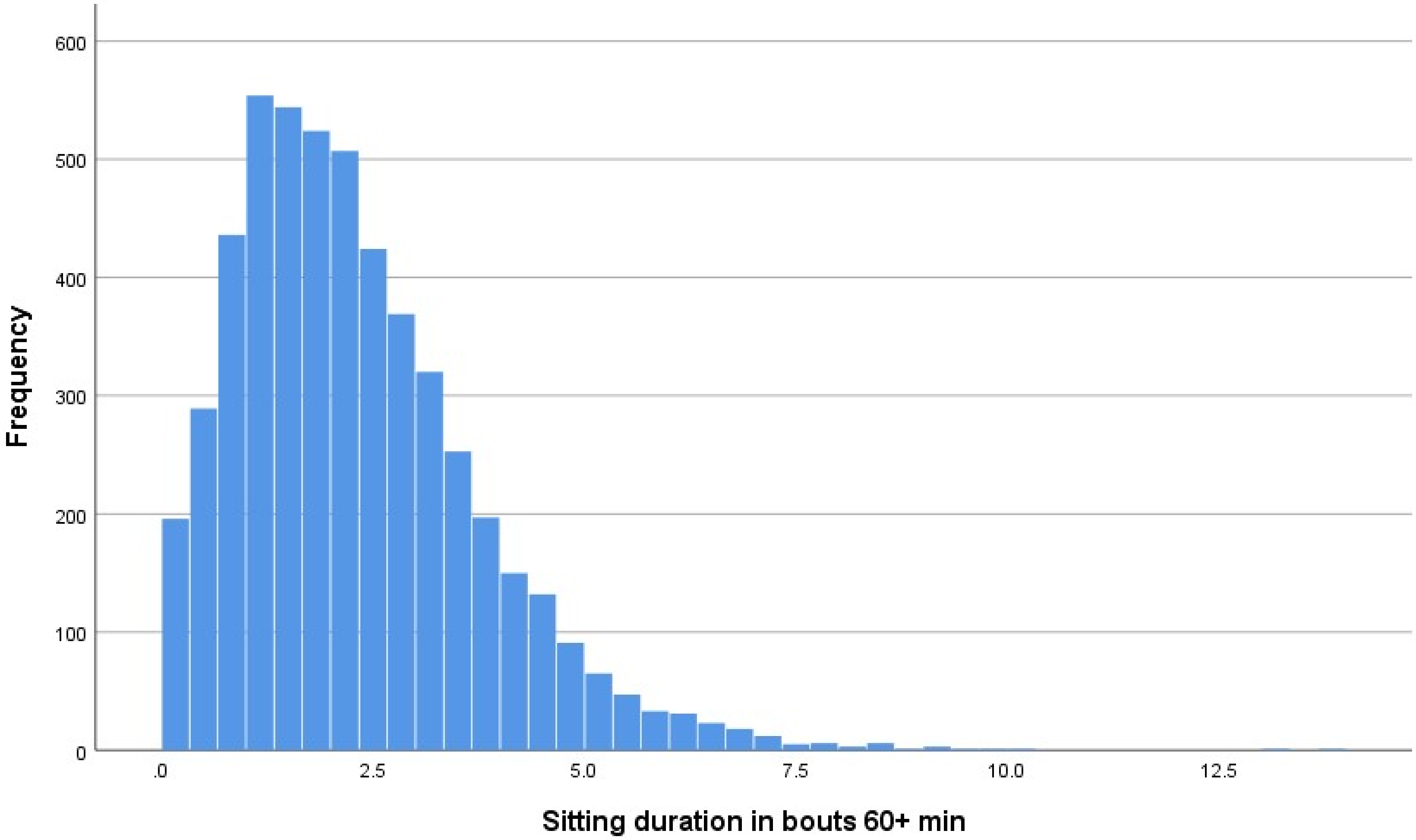

Supplement: kwaa047_Hamer_Web_Material_Final [file kwaa047_hamer_web_material_final.pdf]
